# The Use of Soil Moisture and Pore-Water Pressure Sensors for the Interpretation of Landslide Behavior in Small-Scale Physical Models

**DOI:** 10.3390/s22197337

**Published:** 2022-09-27

**Authors:** Josip Peranić, Nina Čeh, Željko Arbanas

**Affiliations:** Faculty of Civil Engineering, University of Rijeka, 51000 Rijeka, Croatia

**Keywords:** physical modelling, landslides, rainfall infiltration, hydraulic monitoring, pore-water pressure, soil moisture, tensiometers, soil moisture sensors

## Abstract

This paper presents some of the results and experiences in monitoring the hydraulic response of downscaled slope models under simulated rainfall in 1 g. The downscaled slope model platform was developed as part of a four-year research project, “Physical modeling of landslide remediation constructions’ behavior under static and seismic actions”, and its main components are briefly described with the particular focus on the sensor network that allows monitoring changes in soil moisture and pore-water pressure (pwp). The technical characteristics of the sensors and the measurement methods used to provide the metrics are described in detail. Some data on the hydraulic and mechanical responses obtained from the conducted tests on slope models built from different soil types under different test conditions are presented and interpreted in the context of rainfall-induced landslides. The results show that the sensor network used is suitable for monitoring changes in the soil moisture and pwp in the model, both in terms of the transient rainfall infiltration through partially saturated soil and in terms of the rise in the water table and pwp build-up under fully saturated conditions. It is shown how simultaneous monitoring of soil moisture and pwp can be used to reconstruct stress paths that the monitored points undergo during different test phases. Finally, some peculiarities related to hydraulic hysteresis and surface erosion that were observed in some of tests are discussed, as well as possible difficulties in achieving and maintaining the targeted initial moisture distribution in slope models.

## 1. Introduction

Landslides are one of the most common geological hazards. Along with earthquakes, intense or prolonged rainfall is the most common cause of landslides. With ongoing climate change affecting the frequency and intensity of meteorological extremes, which are closely related to the frequency and magnitude of landslides, and the lack of suitable natural environments for the continued expansion of cities and the development of accompanying infrastructure, the increasing interaction between humans and landslides is inevitable. It is therefore no coincidence that the issue of rainfall-induced landslides has received increasing attention from both the landslide scientific community and landslide practitioners in recent decades [1,2,3,4,5,6,7,8,9,10,11].

Despite considerable efforts and advancements in understanding of the physical processes and mechanisms responsible for landslide initiation due to rainfall infiltration [6,11,12,13,14,15,16,17,18,19], the issue remains a major concern for the landslide scientific community. At the same time, many researchers are emphasizing the need for faster implementation of new knowledge into engineering practices, as the gap between the state of the art and the state of the practice continues to widen. Some of the aspects that contribute to the complexity of the landslide triggering problem are: (i) complex physical processes controlling the hydro-mechanical (HM) response of soil in slopes during transient infiltration of rainfall; (ii) highly nonlinear material properties and spatial variations of soils through which infiltration occurs; (iii) quantification of time-dependent boundary conditions, especially at the soil-atmosphere interface, where multiple processes such as infiltration (positive downward movement of water through the soil), surface runoff, evaporation and/or evapotranspiration can occur simultaneously; (iv) hydraulic hysteresis exhibited by the soil depending on the direction of water flow through a soil and the associated effects on the hydraulic and mechanical behavior of the soil; and (v) strong spatial and temporal variations in rainfall characteristics.

Various approaches have been successfully applied to investigate the hydraulic and mechanical response of slopes exposed to different rainfall conditions and the mechanisms leading to the initiation of rainfall-induced landslides. For example, numerical modelling has been successfully used to investigate how different factors affect the stability of both shallow (e.g., [1,7,8,10,16,17,20,21,22,23,24,25,26,27,28,29,30,31,32,33,34,35,36,37,38,39,40,41,42]) and deep-seated landslides under different rainfall conditions (e.g., [19,43,44,45,46,47,48]). Field monitoring has been successfully used to observe the hydraulic and mechanical performance of slopes in different geological and climatic contexts (e.g., [7,17,21,22,31,49,50,51,52,53,54,55,56,57]). Particularly valuable data have been obtained from field experiments conducted on instrumented slopes subjected to increasing soil moisture conditions using rainfall simulators or controlled water inflows from trenches (e.g., [24,42,51,56,57,58,59,60]). Various laboratory devices have been successfully used to observe the HM response of intact or remolded soil samples undergoing conditions representative of soil elements on a slope during the rainfall infiltration (e.g., [61,62,63,64]).

While size-effects, problems associated with the collection and testing of intact soil samples, and limitations in fully reproducing in situ conditions and stress paths are often emphasized as major shortcomings of laboratory sample-scale experiments, the interpretation of data from large-scale field experiments can be hampered by complex slope profile conditions, the introduction of preferential pathways for water flow into a soil, difficulties in installing soil moisture and pore-water pressure sensors, obtaining good hydraulic contact between the sensors and the surrounding soil, etc. Testing downscaled or full-size physical landslide models is another useful approach that, depending on the experimental setup, allows accurate observation of the hydraulic and/or mechanical responses of a slope under precisely controlled initial and boundary conditions. In combination with advanced monitoring techniques and appropriate sensor networks, they have been successfully applied to investigate, for example, the infiltration process, landslide initiation, as well as the propagation of landslides triggered by artificial rainfall under 1 g conditions (e.g., [2,14,65,66,67,68,69,70,71,72,73]). Properly designed physical landslide models have thus proven to be a valuable tool for the accurate measurement of quantities and variables driving the instability phenomena of rainfall-induced landslides. They provide high-quality data that can be used simultaneously for validation and calibration of analytical solutions and numerical models.

This paper encompasses the results from a four-year research project, “Physical modeling of landslide remediation constructions’ behavior under static and seismic actions (ModLandRemSS)”, which started in October 2018 at the University of Rijeka, Faculty of Civil Engineering, Croatia. Firstly, a newly developed platform that enables investigation of small-scale slope models initiated by controlled infiltration of simulated rainfall under 1 g conditions is briefly described, as well as its main components, including the rainfall simulator and the equipment for monitoring the HM response of the slope model during the tests. Particular attention is paid to the sensor network that allows monitoring changes in soil moisture content and pore-water pressure in models exposed to different conditions of simulated rainfall. The main characteristics of the sensors and measurement methods used to provide the metrics are described in more detail. The results of the hydraulic and mechanical responses obtained from various tests on the slope models built from different soil materials and under different test conditions are presented and interpreted in the context of rainfall-induced landslides. Finally, the results presented, and the experience gained during the various activities of the project are used to outline the main advantages and limitations of the chosen approach for monitoring the hydraulic response of physical landslide models. It also outlines some specific features in the measurement results and possible applications of the approaches adopted in this study for investigating specific problems related to rainfall-induced landslides and studying the behavior of unsaturated soils under different loading conditions.

## 2. Platform for Testing Downscaled Slope Models under 1 g Rainfall Infiltration Conditions

One of the main objectives of the project was the development of platforms that enable the investigation of small-scale physical landslide models under static (rainfall) and seismic (earthquake) conditions. The basic idea of the project was to compare the responses of downscaled slope models built from different soil types, geometric conditions and with or without remediation measures applied, while exposed to different loading conditions. It was expected that the results obtained will provide useful data that can be used to predict the behavior of real-scale slopes and improve design procedures and the selection of appropriate remediation measures.

The physical platform considered in this study was designed to allow the initiation of downscaled slope models built from different materials and installation conditions under different rainfall conditions. A network of miniature pore-water pressure and soil moisture sensors allowed observation of the hydraulic response of the scaled models, while the appropriate photogrammetric equipment and other non-contact measurement techniques allowed monitoring of the evolution of the displacement field during testing. Some of the most important parts of the platform are briefly described in the following text, while a detailed description can be found in [72].

**Figure 1:**
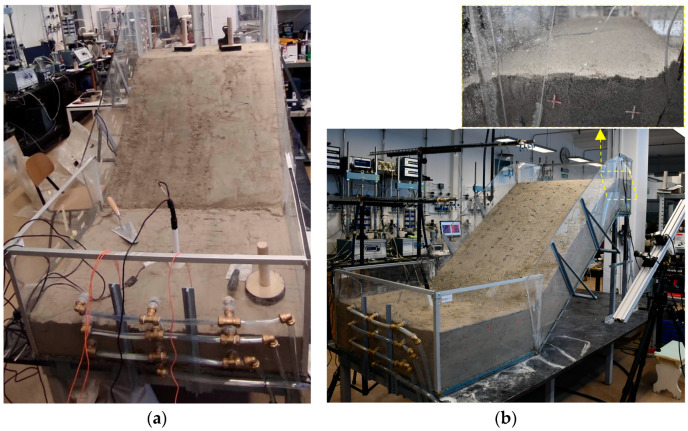
Some details of the model build-up: (**a**) compaction of the soil material with simultaneous installation of the measuring sensors; (**b**) scaled slope model with installed measuring equipment before the start of the test.

The platform itself consisted of steel elements and transparent plexiglass sidewalls that allowed the progress of the wetting front and the onset displacements to be observed during the test. Three basic steel segments, 1 m wide and 0.3 m, 1.4 m and 0.8 m long, formed the upper, middle and lower parts of the platform, respectively. Joint connections and the adjustable height of the upper section allowed the construction of models with different inclinations, while the geogrid attached to the base of the platform prevented the slope material from slipping when in contact with the impermeable steel plates. Liquid rubber and silicone were used to seal the gaps between the plexiglass sidewalls or the perforations in the structure, to ensure that the model was watertight during the experiment. A system of drainage elements inserted through the plexiglass in the bottom of the model allowed the groundwater level (GWL) to be controlled during the test and to be drained after the test was completed. Figure 1 shows the platform during the construction phase of a slope model with the installation of the sensors (a) and the built model before the test with the previously mentioned details (b).

### 2.1. Slope Model Construction and Soil Properties

The platform was suitable to accommodate any soil type and installation at any predefined conditions in terms of the initial density and moisture content. Any of the appropriate standard laboratory methods that are commonly used for installation of soil samples in conventional testing devices (e.g., dry tamping, moist tamping, dry pluviation and wet pluviation) could be used to build-up the model.

The results presented in this study were obtained on three different soil materials as described in the following text. A uniformly graded fine sand (S) was used as a base-soil type and the simplest soil material. It was installed at 50% of the relative density and with 2% of the initial moisture content. Another two types of soil were obtained by adding 10% and 15% of kaolin type clay by weight to the clean sand (from now on denoted as SK10 and SK15, respectively). While SK10 was installed at 50% of the relative density and 5% of the initial water content, relative density of 75% and 8.1% of the initial water content were selected for the SK15 soil. For all soil materials, Ladd’s under-compaction method [74] was used to build slope models 30 cm high, by compacting 5 soil layers of 6 cm height and trying to achieve as homogeneous conditions as possible, in terms of the density and soil moisture distribution. After the soil model was built, the entire model was covered with nylon to prevent excessive drying due to evaporation till the start of a test. Basic soil properties of soils used in the study are reported in Table 1.

### 2.2. Rainfall Simulator

In addition to the HM properties of the soil, rainfall characteristics play a decisive role in rainfall-induced slope failures. The design of a rainfall simulator that met the specific requirements of the project was an important issue in the early stages of the platform’s development. In particular: the ability to apply a wide range of rain intensities depending on the soil material tested and the specific objectives of the experiment, sufficient spatial uniformity of the simulated rainfall and the ability to change rainfall patterns and characteristics, the prevention of excessive erosion due to raindrop impact forces (which are closely related to raindrop diameter and impact velocity), the portability of the rainfall simulator, due to its possible use not only with the platform for testing under static conditions, but also under dynamic conditions, and the limited budget available for its construction were the main considerations in the design and construction of the simulator.

**Figure 2:**
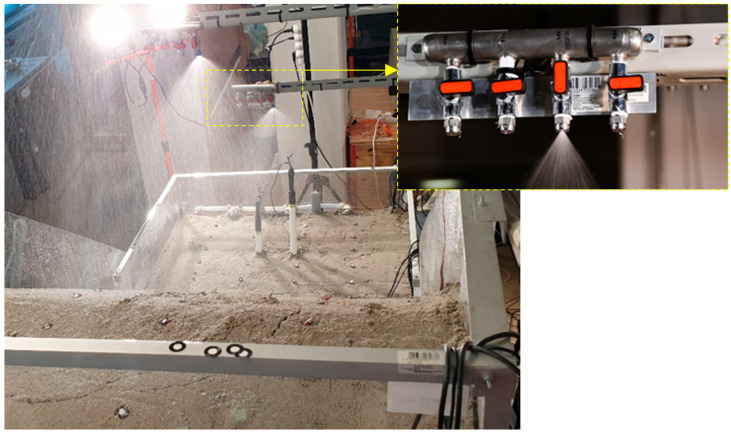
Simulated rainfall in the test on SK10 with 40-degree angle of inclination (I = 23 mm/h) with a detail of the sprinkler branch equipped with four axial-flow full cone nozzles.

The rainfall simulator developed consisted of three independent branches that delivered water from the main control block to the spray nozzles. The main control block was connected to a water supply network and consisted of the control units that regulated the rainfall simulator’s work, such as water pressure regulators, manometers, flow measuring units, filters, etc. High-density polyethylene pipes conveyed the pressurized water from the control block to three sprinkler branches, which were adjustable in height and equipped with various spray nozzles (Figure 2). At a reference pressure of 2 bar, the axial-flow full-cone nozzles (Table 2) could generate rainfall intensities ranging from less than 30 mm/h to more than 150 mm/h. The possibility of implementing a system of electromagnetic valves and relays allowed the automation of the system, which could be easily modified to accept any desired rainfall intensity or pattern. The rainfall simulator was fully portable and could be easily dismantled and installed at any location with a water supply ensured. However, on-site use would require the use of pumps in addition to the water supply, which should be integrated into the simulator unit.

### 2.3. Monitoring Equipment

The monitoring equipment can be divided into two main parts: (i) a geodetic monitoring system based on innovative photogrammetric equipment for multi-temporal landslide analysis [75] of image sequences obtained from a pair of high-speed stereo cameras [76], terrestrial laser scanning, and structure-from-motion (SfM) photogrammetry surveys, allowing the determination of the surface model of the slope in the pre- and post-failure phase [77]; (ii) geotechnical monitoring system consisting of a network of miniature sensors to measure changes in soil moisture, pore-water pressures, soil temperature and electrical conductivity, displacements, etc.

**Figure 3:**
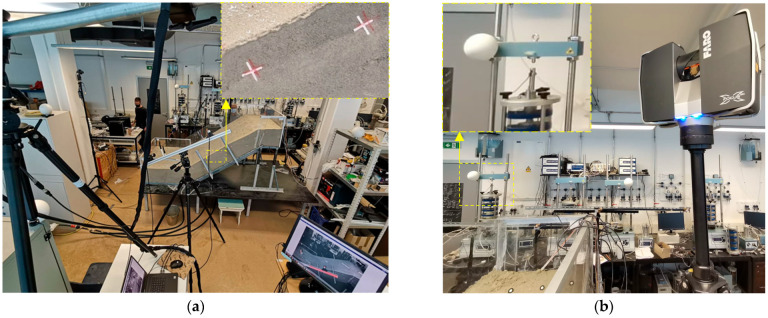
Geodetic monitoring equipment: (**a**) A view of the model from the ARAMIS measuring system (GOM mbH, Braunschweig, Germany) and a detail from the (front) 12-megapixel camera monitoring displacements through the plexiglass wall; (**b**) The FARO Focus 3D X 130 (FARO Technologies Inc, Lake Mary, USA) terrestrial laser scanner and a detail of the reference sphere location.

The ARAMIS is an optical, noncontact 3D measurement system (GOM mbH, Braunschweig, Germany) that provides the entire workflow from taking measurements to analyzing data and presentation of the results. A set of two 4-megapixel and two 12-megapixel high-speed cameras were used for multi-temporal landslide analysis from captured stereo image sequences. The system enables continuous monitoring of the 3D coordinates of reference points through all stages of the activity of scaled landslide models. The FARO Focus 3D X 130 (FARO Technologies Inc, Lake Mary, FL, USA) terrestrial laser scanner was used to capture the 3D model surface at high resolution before the start and at the end of the test. The scanner uses phase-shift technology to accurately determine the relative 3D position of the scanned points, while reference points are used to overlap the scanned areas and define the relative coordinate system. A Nikon D500 camera (Nikon Inc, Melville, NY, USA) with an ultra-wide-angle Tokina AT-X 11–20 lens was used for the SfM photogrammetry survey of the physical models. This technology allows the creation of 3D models from multiple overlapping images taken at different triangulation angles. Figure 3 shows the aforementioned equipment, while a detailed technical specification can be found in [72].

The geotechnical monitoring system consisted of a complex network of miniature sensors equivalent to the equipment used for field monitoring. All sensors were connected to data loggers, enabling continuous data collection on soil moisture, positive pore-water pressures and soils suction, electrical conductivity, temperature, pressures, displacements, and accelerations. As the focus of this presentation is on the role of hydraulic monitoring of scaled slopes, the measuring devices are described in more detail in the following section.

## 3. Equipment for Monitoring the Hydraulic Response of Slope Models

Accurate observation of the HM response of scaled soil slopes under precisely controlled initial and boundary conditions provides valuable information on quantities and variables controlling the instability phenomena of rainfall-induced landslides. The development of a suitable sensor network for the test conditions and the selection of appropriate measurement techniques is therefore a crucial step in the development of small-scale physical landslide models.

The following part describes the main components that made up the hydrological monitoring system of the physical model. There were several requirements that were considered during the selection of the measuring devices. Firstly, information on soil moisture content and the change in pore-water pressure (both positive and negative with respect to atmospheric pressure) during rainfall infiltration had to be available at appropriate time intervals, depending on the experimental conditions and the variable under consideration. This requirement necessitated the use of digitized sensors and pressure transducers in combination with suitable data loggers with programmable data collection intervals. The second important requirement that had to be taken into account was that the project envisaged testing a wide range of soils, from sand to clay. Therefore, the selected measurement equipment had to be able to cover a wide measurement range occurring in different soil textures, while ensuring sufficient precision and resolution of the measured quantities, as well as the reasonable responsiveness and equilibration times depending on the type of soil tested. The solution was found in the soil water content sensors, which use the capacitance method to predict volumetric water content in soil [78], allowing rapid detection of changes in soil moisture content for a wide range of soil textures, porosities and density conditions. In sandy soils or soils where suction values within the measuring range of standard tensiometers are to be expected, mini-tensiometers are used for rapid measurement of both pore-water pressure and suction. Soil water potential sensors equipped with porous ceramic discs and the dielectric permittivity measurement method were intended for measurements in fine-grained soils with relatively low hydraulic conductivity, such as clay, where high matric suction values are to be expected. Another consideration relates to data redundancy: data had to be collected simultaneously at several depths and along different profiles in order to obtain a complete picture of the temporal and spatial evolution of different variables during the experiment. Finally, the chosen monitoring equipment was intended to be used with both the small-scale physical landslide models for testing under static (rainfall) and seismic (earthquake) conditions.

### 3.1. Soil Water Content Sensors

The TEROS 10 and TEROS 12 are soil moisture sensors from the METER Group AG, Munich, Germany for indirect measurement of the volumetric water content of soils or other porous materials. Both sensors are based on the capacitance method for predicting the amount of water in the soil based on the electrical properties of the soil and the calibration procedure proposed by Topp in 1980 [78]. An electromagnetic field (70 MHz oscillating wave) is used to measure the apparent dielectric permittivity (*ε_a_*) of the soil. The sensor supplies an oscillating wave to sensor needles which charge according to the dielectricity of the material. The charging time is proportional to the dielectricity and to the VWC of the substrate [79,80]. Depending on the measured charging time, a microprocessor outputs a raw sensor value (*RAW*) based on the substrate *ε_a_*. Finally, a calibration equation specific to the substrate is used to convert the values from *RAW* to VWC. The high measurement frequency ensures insensitivity to variations in soil texture and electromagnetic conductivity (EC) [79]. In all tests, a generic calibration equation from the manufacturer for mineral soils was used to predict VWC, based on the *RAW* output from the METER data logger ZL6. For TEROS 10 it reads, for example [80]:

Θ = 3.879E(−4) × *RAW*–0.6956 (1)

while the *ε_a_* can be used to determine VWC, e.g., using the Topp’s equation [78]:

*ε_a_* = (2.887E-9x(*RAW*)E3–2.080E-5x(*RAW*)E2 + 5.276E-2x(*RAW*)–43.39)^2^ (2)

**Figure 4:**
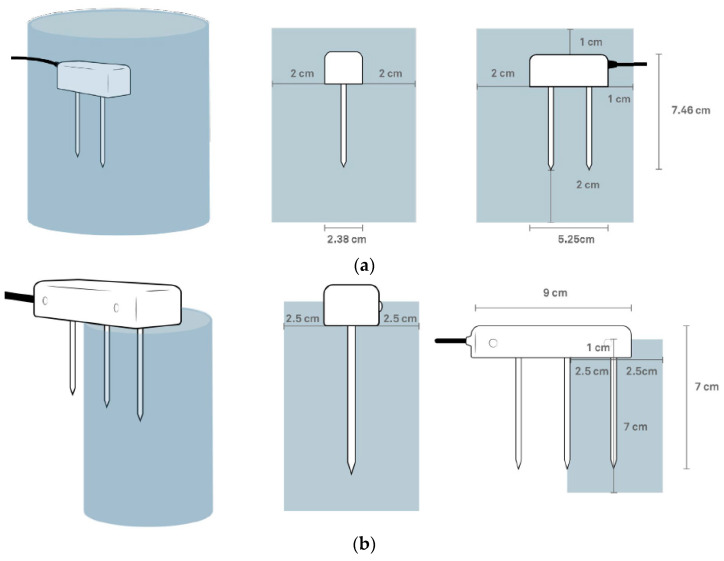
Volume of influence for: (**a**) TEROS 10 and (**b**) TEROS 12 sensors (modified from [81,82]).

**Figure 5:**
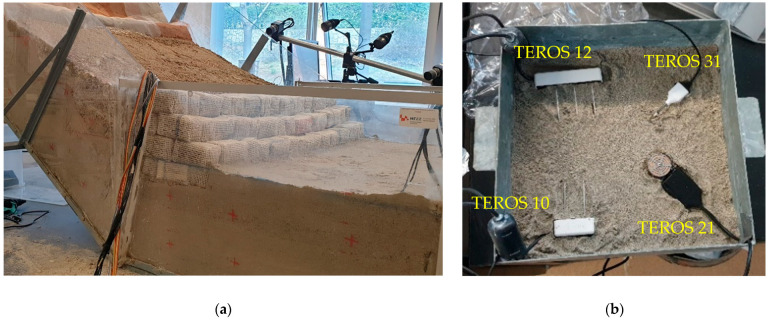
Details on (**a**) model setup with installation of sensors and cameras and (**b**) TEROS 12, 10, 31 and 21 sensors (top and bottom left and right, respectively) for hydraulic monitoring of slope models during the calibration procedure.

TEROS 10 has a VWC range for mineral soils of 0.00–0.64 m^3^m^−3^ with a resolution of 0.001 m^3^m^−3^ and an accuracy of ±0.03 m^3^m^−3^ in mineral soils with a solution EC < 8 dS/m [80]. On the other hand, TEROS 12 has a slightly larger VWC measurement range (0.00–0.70 m^3^m^−3^) with the same resolution and accuracy as TEROS 10. The temperature range for TEROS 12 is −40 to 60 °C, with a resolution of 0.1 °C and a measurement accuracy of ±1 °C. The bulk electrical conductivity measurement range of TEROS 12 is 0–20 dS/m with a resolution of 0.001 dS/m and a measuring accuracy of ±3% [79]. The outer needle and the needle in the middle are used to measure VWC, while the inner needle, which is closest to the ferrite core, and the needle in the middle are used to measure EC of the soil. The TEROS 12 has a measurement sensitivity volume of 1010 mL, which is significantly larger than the 430 mL sensing volume of the TEROS 10 (Figure 4). Figure 5 shows some details on the installation and calibration of the sensors.

Given the limited dimensions of downscaled physical models, sensing volumes can often significantly affect measurement results and must be taken into account when selecting measurement equipment and determining measurement locations. The direction and type of installation of the sensor must also be considered. Although a vertical installation usually ensures a larger volume of influence and thus more representative values, e.g., in heterogeneous soils and in-situ applications, for laboratory measurements on relatively homogeneous slope models, such as those considered in this study, an installation with needles placed parallel to the soil layers can provide more accurate information on the wetting front advancement, the rise of the water table and changes in soil moisture conditions generally at discrete depths, i.e., at the measurement points. In addition, when installing the sensors, care must be taken with the routing of the cables (and in particular the large ferrite core in the case of the TEROS 10 and 12 sensors) to avoid creating preferential pathways for water flow or affecting the failure mechanism of the slope model.

Therefore, both TEROS 10 and 12 were installed parallel to the soil layers after compaction was complete, with a minimum vertical distance of 6 cm along the measurement profile to avoid interference with the measurement. The cables were laid horizontally during the build-up of the models and routed laterally to the lower part of the model to minimize the influence on the hydraulic and mechanical response of the slope models. To minimize interference with the non-contact optical measurement systems, the cables were bundled and routed out on the opposite side of the camera systems, behind the existing rods and steel elements (Figure 5a).

### 3.2. Soil Water Potential Sensors

Depending on the activities and the funds available, standard tensiometers with vacuum gauges (IR −45 and T1) and digital (TEROS 32) tensiometers, mini tensiometers (TEROS 31) and TEROS 21 soil water potential sensors were purchased at a certain stage of the project development to monitor changes in soil water potential values. The non-digitized standard tensiometers IR −45 (Irrometer Company Inc, Riverside, CA, USA) and T1 (MMM Tech Support GmbH & Co. KG, Berlin, Germany) were already purchased in the initial phase of the project and used for the first test, which was carried out on a slope model in clean sand with a 30-degree inclination. In addition to good hydraulic contact of the ceramic with the surrounding soil, which ensures measurement accuracy, all tensiometers must be appropriately conditioned before insertion into the soil to achieve full measurement range and rapid response during the equilibration phases. This treatment is often referred to as “preconditioning”. TEROS 32 and TEROS 31 digitized tensiometers (METER Group AG, Munich, Germany) were procured in the later stages of the project and were used with METER’s ZL6 data loggers to continuously collect measurements at time intervals of up to 1 min in other soil materials, i.e., SK10 and SK15. The TEROS 21 (METER Group AG, Munich, Germany) sensors were intended to be used in a measuring range that exceeds that of standard (mini) tensiometers. At a certain point of the development of the project, standard tensiometers with vacuum gauge (IR-45 and T1) and digital (TEROS 32) tensiometers, mini-tensiometers (TEROS 31), and TEROS 21 soil water potential sensors were acquired for measuring the soil water potential purposes, based on the project requirements and funds available. Non-digitalized, standard IR-45 (Irrometer Company Inc, Riverside, CA, USA) and T1 (MMM Tech Support GmbH & Co. KG, Berlin, Germany) tensiometers were obtained in the very early stage of the project and were used in the first test conducted on clean-sand. Digitalized TEROS 32 and TEROS 31 tensiometers (METER Group AG, Munich, Germany) were obtained in the later stages of the project and were used with METER’s data loggers ZL6 to continuously collect measurements at time intervals up to 1 min in other soil materials. The TEROS 21 (METER Group AG, Munich, Germany) sensors were intended to be used in the measurement range exceeding that of standard (mini) tensiometers.

TEROS 21 is a maintenance-free matric potential sensor designed for long-term, continuous field measurements [81]. TEROS 21 uses a similar approach as the VWC sensors TEROS 10 and TEROS 12: The sensor measures the dielectric permittivity of a solid matrix, i.e., porous ceramic discs, to determine their water content. Since the dielectric permittivity of porous ceramic discs is highly dependent on the amount of water present in the pore spaces and the porous ceramic discs tend to reach hydraulic equilibrium with the surrounding soil, the measured water content of the solid matrix is used to determine the water potential of the soil based on a known soil water retention curve (SWRC) of porous stones. The ceramic discs are manufactured to cover a wide water potential range, i.e., they have pores with different radii: from larger ones related to wet-end limitations to the smallest ones responsible for dry-end measurements. The main limitations in the dry-end measurement range are due to measurement inaccuracies caused by high changes in water potential with small changes in measured water content. On the other hand, the air entry value (AEV) of the largest pore in ceramics (−9 kPa for gen1 and −5 kPa for gen2 sensors) may limit measurements in soils with lower AEVs, where desaturation of porous discs only starts at lower water potentials [81].

**Figure 6:**
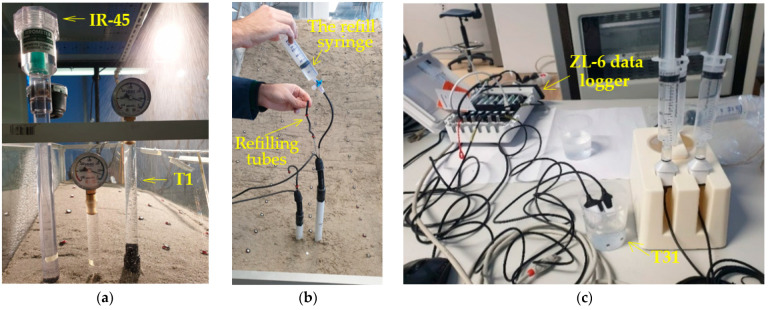
Standard, non-digitalized IR and T1 tensiometers in tests on clean sand (**a**); preconditioning of TEROS 32 (**b**); and TEROS 31 tensiometers (**c**).

With an active surface of only 0.5 cm^2^ and a diameter of 5 mm [82], TEROS 31 is the soil water potential and temperature sensor that is particularly suitable for laboratory tests, such as soil column or SWRC tests. The sensor has a water potential measurement range of −85 kPa (or up to −150 kPa during boiling retardation) to +50 kPa with a resolution of 0.0012 kPa and an accuracy of 0.15 kPa, while the temperature measurement range is from −30 to +60 °C, with a resolution of 0.01 °C and an accuracy of 0.5 °C [81]. The TEROS 32 offers the same possibilities in terms of the measuring range, resolution and accuracy for both water potential and temperature. However, with a diameter of 2.5 cm and a length of 40 to 120 cm, low power requirements and the ability to refill the sensor externally without having to remove it from the ground [83], TEROS 32 is a sensor primarily designed for long-term, continuous field measurements. The ability to refill TEROS 32 sensors proved very useful in long-term tests on sand-kaolin mixtures when residual water conditions were achieved over extended periods without rainfall simulation. The low disturbance to the soil and ease of installation during the build-up of the slope models, as well as the particularly fast response, were some of the best features of the TEROS 31 sensors found for the application considered in the study. Given the robustness of the TEROS 32 sensors, they could only be installed in locations where no interaction with the failure mechanisms was expected, such as the lower part of the model (Figure 6b). Some details on the measurement and conditioning of the sensors can be found in Figure 6.

## 4. Examples of Measurement Results and Data Interpretation

This section presents some of the data obtained with the HM slope model monitoring equipment in different experimental setups: i.e., using different soil materials, geometric conditions and simulated rainfall characteristics. The results are interpreted in the context relevant to a study of rainfall-induced landslides, with particular attention on the specificities associated with downscaled physical landslide models. Some interesting details from four different experiments are singled out and discussed in more details.

### 4.1. Example 1: Initiation of Sandy Slope due to GWL Rise

**Figure 7:**
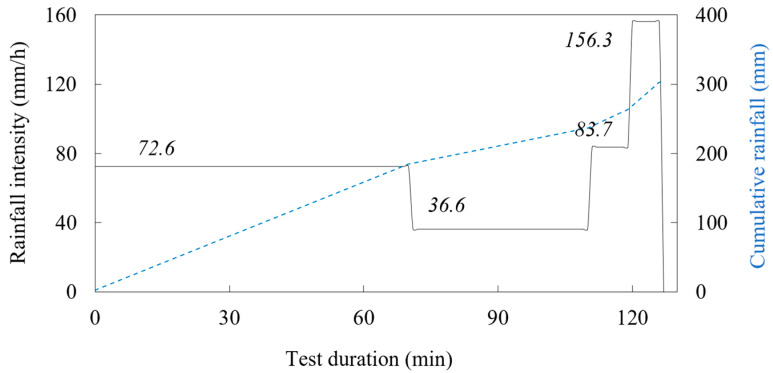
Simulated rainfall in the test considered in Example 1.

**Figure 8:**
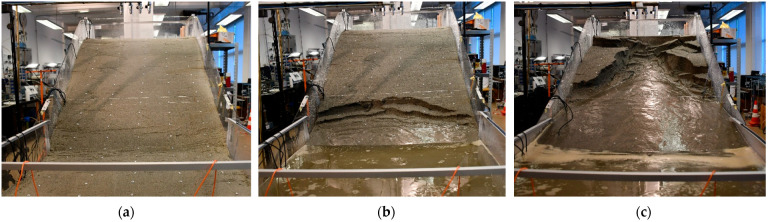
The sandy slope model considered in Example 1 at the beginning of the test (**a**); after 77 min of rainfall simulation (**b**); and at the end of the test (**c**).

The first example considers the model built in uniform sand with a slope angle of 35 degrees and subjected to a constant rainfall intensity of 72.6 mm/h. The slope, built by compacting five layers of soil with a moisture content of 2% to a relative density of 50%, remained stable for the first 56 min of the test, when the first instability occurred in the form of a small rotational slide at the slope’s toe. Thereafter, further instability developed in the form of retrogressive slides that reached the top of the slope in a relatively short time compared to the duration of the pre-failure phase. 60 min after the onset of rainfall, the highest drainage pipe was opened to maintain the GWL level at the soil surface in the lower part of the model, while in the 72nd minute the L-branch of the rainfall simulator was closed as the entire lower part of the model was flooded. In the 96th minute of the test, the middle and lower drainage pipes were also opened to facilitate outflow from the model and maintain the GWL at the soil surface in the lowest section of the model. As the retrogressive slides progressed towards the upper part of the slope model, the simulated rainfall was increased in the upper parts of the model to accelerate the development of instability until the 127th minute of the test when the end of the test was declared, and the rainfall stopped. The simulated rainfall from the test considered in Example 1 is summarized in Figure 7, while the overview of the test in terms of instability development from the photographs taken during the test is given in Figure 8.

**Figure 9:**
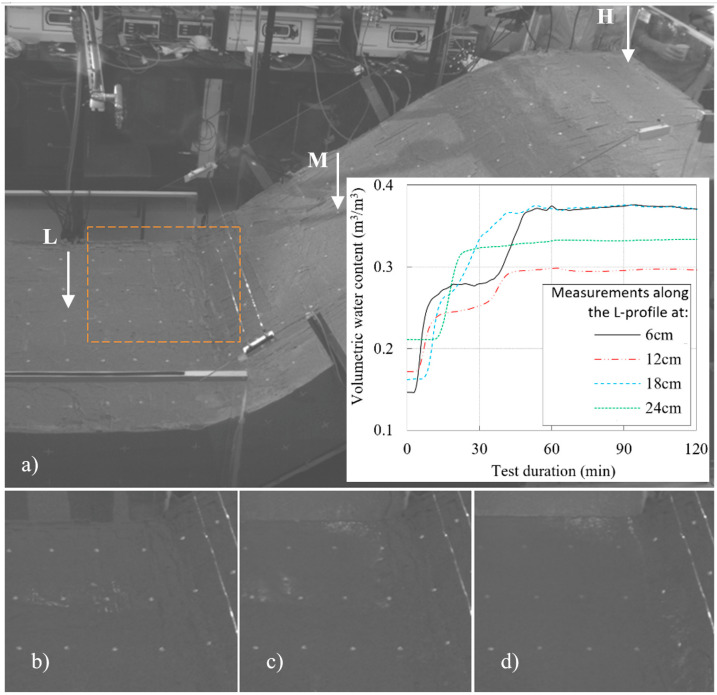
Model from the Example 1 as captured by the system ARAMIS (GOM GmbH): (**a**) at the beginning of the test (with indication of the measurement profiles and VWC changes measured at the L-profile at 6, 12, 18 and 24 cm depth; (**b**) 49th minute of the test with first traces of GWL reaching the surface in the central part of the base; (**c**) 51th minute of the test with the entire base submerged; and (**d**) 56th minute of the test with contours of the first small rotational landslide visible at the foot of the slope.

Figure 9a shows an image of the model taken with the system ARAMIS (GOM GmbH) at the beginning of the test, indicating the positions of the measurement profiles in the upper (H), middle (M) and lower (L) slope sections. In order to observe changes in soil moisture and GWL conditions at the base of the slope model (i.e., the focus is on the area of the model marked with an orange square), the same figure also embeds VWC measurements collected at 6, 12, 18 and 24 cm depth on the L measurement profile. Some specific moments captured by the optical system ARAMIS that are relevant for reconstructing the onset of instability in the experiment under consideration are shown in the other three figures. For example, Figure 9b shows the model 49 min after the start of the rainfall, when the first trace of GWL reaches the ground surface in the central part of the bottom of the slope. As can be seen in Figure 9c, the entire base is submerged after 51 min of the rainfall simulation, while in the lower right corner of Figure 9d, taken 56 min after the start of the test, the contours of the first small rotational landslide can be seen. Figure 9b–d only show the part of the model marked with the orange square.

The results of the VWC measurements summarized in Figure 9a revealed several interesting points. First, the results suggest that the initial moisture distribution conditions along the L measurement profile was not completely homogeneous. Instead, the VWC seemed to be slightly reduced in the layers close to the surface (e.g., see the VWC sensor at 6 cm depth), while in deeper layers the moisture content was slightly increased (e.g., sensor at 24 cm depth). Shortly after the rainfall starts, the VWC seemed to increase to a certain value, which is lower than the saturated value. The increase in soil moisture was observed in the form of saturation from top to bottom due to the infiltration of the rainfall in the first phase of the test: only 3 min after the start of the rainfall, the increase in VWC was recorded by the shallowest sensor at 6 cm depth. The same was observed for the sensors at 12, 18 and 24 cm depth after 6, 8 and 13 min of the test, respectively. Thereafter, the VWC began to increase further at greater depths (see, for example, the sensor at 24 cm depth), while the further increase in moisture for the sensors at shallower depths occurred with some delay, indicating a quasi-steady state seepage condition for a certain period of the test duration. Finally, the VWC readings became constant at a certain point in the test, first at the deeper measurement points and then also at the shallower ones, indicating that saturated conditions had been reached. According to the measurement results, the GWL reached the soil moisture sensors installed at 24, 18 and 12 cm after about 23 min, 39 and 41 min, respectively. The VWC readings became constant and reached the maximum value corresponding to saturated conditions at a depth of 6 cm, about 48 min after the onset of rainfall. These summarized values correspond exactly to the observations made during the experiment and to the timings given in Figure 9.

The observations from Example 1 indicated that despite certain deviations in the absolute measured values, which could be due to various reasons, such as requirements of the calibration procedure of each sensor, different readings of the TEROS 10 and TEROS 12 sensors, or different soil densities achieved around the moisture probes, or uneven conditions in terms of contact and the presence of gaps between the surrounding soil and the sensor probes, the soil moisture sensors used in the study could provide useful data on the trend of soil moisture increase in general, providing information on the saturation conditions during the infiltration process as well as on the conditions of GWL rise in the later phase of the test.

### 4.2. Example 2: Hydraulic Paths and a Reduction in the Shear Strength due to Rainfall Infiltration

**Figure 10:**
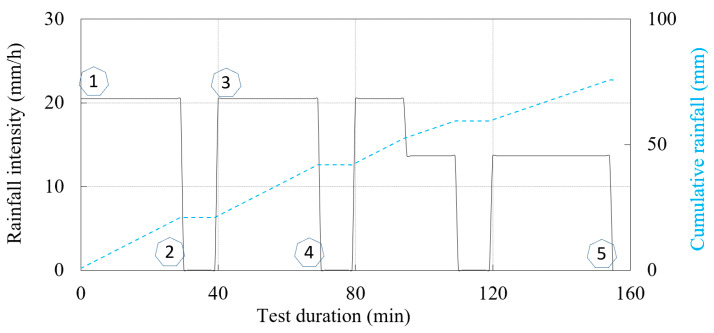
Simulated rainfall in the test considered in Example 2.

**Figure 11:**
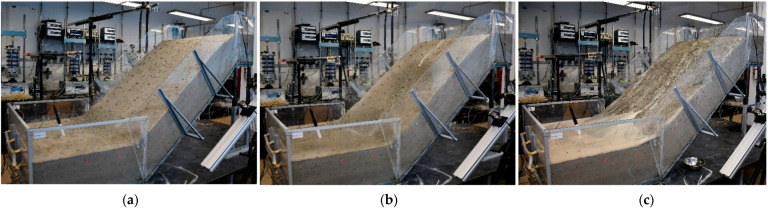
The SK15 slope model considered in Example 2 at the start of the test (**a**); 18 min (**b**); and 159 min after the start of the test (**c**).

The second example deals with the hydraulic paths and stress states that the 40-degree slope model created in SK15 material undergoes during a simulated rainfall. The SK15 material was compacted at a relative density of 75% and a soil moisture content of 8.1% (Table 1), resulting in slightly higher VWC and lower matric suction values at the beginning of the test. In this case, a continuous rainfall (intensity 20.5 mm/h) was interrupted by 10-min breaks to observe the HM response of the model (Figure 10). Traces of surface water ponding in the lower part of the model, i.e., at the foot of the slope, were observed in this test very shortly after the rainfall was started in this test: after only about 6 min. In contrast to Example 1, instabilities in this test mainly occurred in the form of surface erosion, while no shear bands or cracks formed due to the infiltration of the rainfall. An overview of the test in terms of instability development based on the photos taken during the test is provided in Figure 11.

**Figure 12:**
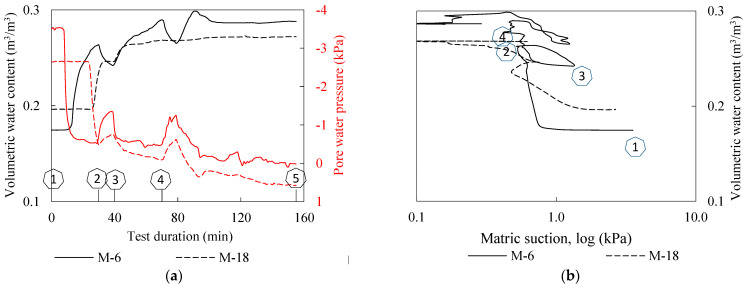
Hydraulic monitoring data from the test considered in Example 2 for points at 6 (M-6, continuous lines) and 18 cm depth (M-18, dashed lines) on the M measurement profile: (**a**) VWC and pore water pressure changes during the test; and (**b**) data in the logarithmic (*u_a_-u_w_*) vs. VWC plane.

Again, the lowest sprinkler branch was closed after the entire lower part of the model was flooded, i.e., after 95 min in this test. The hydraulic monitoring data obtained for two measurement points on the M measurement profile (the same analogy is used as in Example 1—see Figure 9 at 6 (M-6) and 18 cm depth (M-18) are shown in Figure 12. As both the M-6 and M-18 measurement points were instrumented with a pair of TEROS 10 and TEROS 31 sensors, insights into the changes in soil moisture and pore water pressure during the test were available. Figure 12a shows the data on the changes in VWC and pore water pressure during the test, while the same data are shown in Figure 12b in the logarithmic (*u_a_-u_w_*) vs. VWC plane. The numerical markers in the following figures serve as reference points to follow the course of the experiment and the simulated rainfall conditions when the data are presented in different forms and planes. For example, the number “1” denotes the beginning of the experiment, i.e., the start of the rainfall simulation for the first 30 min. The number “2” denotes the end of the first 30min rainfall period and the closure of all spray nozzles for 10 min. The start of the next phase of the test with another 30-min rainfall period is marked with the number “3” and so on. The monitoring data for the first 155 min (denoted with the number “5”) of the test are included in this presentation, after which the data is excluded from the plot.

It is interesting to observe from the data presented in Figure 12 that the suction and VWC changed in a consistent manner, although there were some differences in the peak values and timings for the two monitored points. The TEROS 31 installed at the M-6 location indicated an abrupt decrease in matric suction from the 8th to the 13th minute of the test, where the matric suction dropped from 3.5 kPa to 0.74. From this point on, the pwp condition remained essentially unchanged until the end of the first phase of rainfall simulation “2” (i.e., the 30th minute of the test). TEROS 10 at the same observation point, on the other hand, indicated that the VWC gradually increased, reaching a maximum value of 0.265 at “2”. During the first no-rainfall phase (“2”–“3”), the VWC decreased and the matric suction increased, followed by an increase in VWC and a further decrease in matric suction with the new phase of rainfall infiltration “2”–“3”. A similar trend was observed in the final phase of the test, with matric suction and VWC content approaching zero and constant values, respectively, after the 110th minute of the test, indicating saturated or near-saturated conditions at measurement point M-6. A similar but less pronounced response was observed for the lower monitoring point M-18, but with a time delay of about 15 min compared to point M-6 in the first phase of the test (“1”–“2”). An interesting detail for point M-18 was that although the response to simulated rainfall can be inferred from the soil suction measurements (“2”–“3” and “4”), the reduction in VWC observed for point M-6 during the no-rainfall phase did not appear to be observed for the lower monitoring point M-18, which according to the pwp data was submerged or nearly saturated much earlier in the test (“4”). As seen from the pwp and VWC data in Figure 12a, M-18 reached saturated conditions much earlier compared to point M-6, i.e., about 70 min after the start of the test.

**Figure 13:**
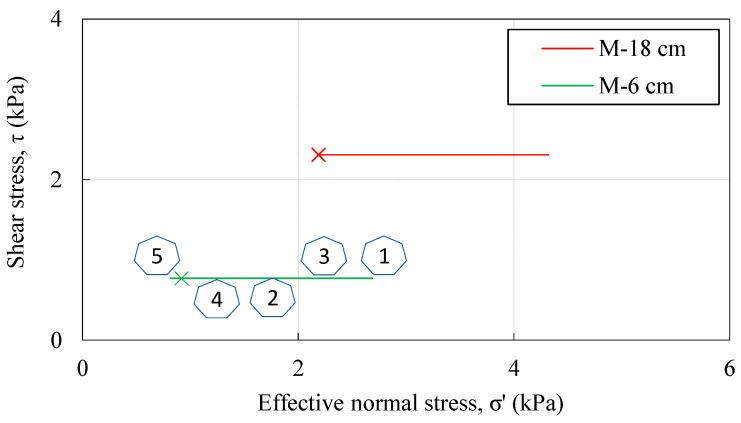
Stress paths calculated using Equation (3) and the data collected in Example 2 for monitoring points along the M-profile at 6 (M-6) and 18 (M-18) cm depth.

The data obtained was used in the following part of the presentation to calculate the changes in effective stress during transient infiltration of rainfall and the effects on the decrease in soil shear strength during the test for the two points monitored (Figure 13). The initial stress state and corresponding shear strength, located in the right side of the plane, reflect the soil moisture conditions at the beginning of the test, which were achieved during the time of model construction and remained mostly preserved until the start of the test. With the onset of rainfall infiltration, there was an increase in VWC and dissipation of the matric suction in the form of the transient process (Figure 12). If one makes the simplified assumption that capillarity is the only retention mechanism, a reduction in effective stress during rainfall infiltration can be quantified from the VWC and pore water pressure values observed during the experiment by using, for example, the equation for effective stress by Bishop [84]:

(3) $$\sigma^{\prime }=\sigma -u_{a}+\chi \left(u_{a}-u_{w}\right),$$

where the effective stress parameter *χ* is set equal to the effective degree of saturation. With a residual volumetric water content of *θ_r_* = 0.05 m^3^ m^−3^ and a saturated volumetric water content *θ_s_* defined using the VWC values measured at full saturation of the monitored points (data in Figure 12a), the result is the plot of the stress paths shown in Figure 13.

A further simplification is that the change in the unit weight of the soil during transient infiltration of rainfall was not considered, but instead a constant weight of 20 kN/m^3^ was used in the analysis. The movement from right to left indicates that the matric suction decreases and the saturation conditions increased. Two crosses on the left side of the stress paths indicate the stress conditions at the beginning of the test calculated according to Terzaghi’s effective stress equation, i.e., without considering the effect of soil suction. This simple example illustrates the importance of considering the principles of unsaturated soil mechanics, as the build-up of the downscaled slope models usually involves moist tamping of the soil under partially saturated conditions. Due to the low confining pressure in such models, the shear strength component associated with the matric suction that develops under partial saturation may thus represent a large portion of the total soil’s shear strength. Although the suction-related shear strength component is very limited in the case considered, as the effect of the already low suction values monitored in the example is further scaled by the effective degree of saturation, the effect on the total available shear strength is nevertheless not negligible, considering that the monitored points are located at 6 and 18 cm depth. The situation could be quite different for soils with high AEVs, where relatively high matric suction can develop under near-saturated conditions. The experimental setup chosen in this study to monitor the hydraulic response appears to be very useful for investigating and quantifying these aspects.

Another interesting point about the simultaneous use of soil moisture and pore water pressure sensors at the same monitoring point of the slope model is evident from the plots of the hydraulic paths in Figure 12b. The results obtained indicate that both monitoring points may have been affected by hydraulic hysteresis effects during the simulated rainfall. Recent studies have shown that this phenomenon can have a major impact on the HM response of the soil and on the stability of a slope during rainfall in general (e.g., [17,85,86]). While most studies that investigated hydraulic hysteresis were based on the results of various laboratory devices or instrumented slopes in situ (e.g., [31,87,88]), the results obtained in this study suggest another possible useful application of the small-scale physical landslide models. Despite the obvious disadvantages in terms of limited dimensions and differences compared to measurements on in situ instrumented slopes, such slope models offer advantages in terms of controllable geometric, initial and boundary conditions, as well as the implementation of specific vegetation types, remediation measures, etc. in experiments.

### 4.3. Example 3: Initial Soil Moisture Distribution and the Instability Type: Erosion vs. Sliding

The third example addresses the issue of the build-up of the downscaled slope models at the target water content and discusses different types of instability observed as a function of the initial soil moisture content, which is often related to the preceding rainfall conditions. These issues are discussed based on the results presented in the following part and some general experiences of the authors from different activities within the project.

**Figure 14:**
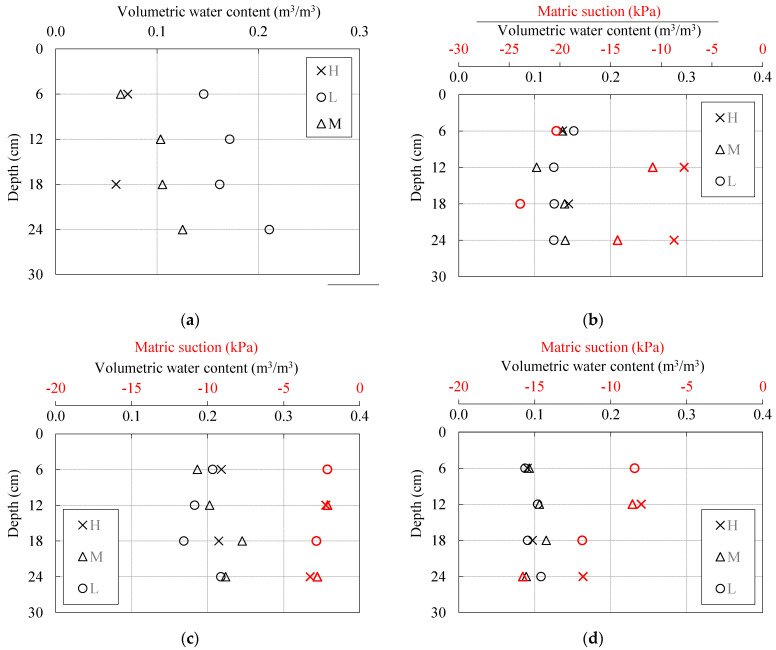
Initial volumetric water content and matric suction distributions along the upper (H), middle (M) and lower (L) measurement profiles for tests on 35-degree inclined slope models built in: (**a**) clean sand; (**b**) SK15; and SK10 with higher (**c**) and lower initial soil moisture contents (**d**).

Figure 14 shows the data on the distributions of soil moisture and pore water pressure at the beginning of the test (i.e., immediately before the start of the rainfall) in models constructed of different soil materials. The initial soil moisture distributions for the slope considered in Example 1 (model constructed from clean uniformly graded sand with a slope angle of 35 degrees) for all three measurement profiles (see Figure 9a) are shown in Figure 14a. Since all models built in SK10 and SK15 were also equipped with TEROS31 sensors, the data on initial matric suction are also included in Figure 14b–d). Figure 14b shows the initial conditions for a slope made of SK15 material with an inclination of 35 degrees, while Figure 14c,d show the same for slope models made of SK10 material at the same inclination of 35 degrees. The difference between the models in Figure 14c,d is only that the model in Figure 14c was built with a higher initial moisture content.

Although care was taken to create conditions as homogeneous as possible in terms of density and soil moisture when creating the slope models, the data presented in Figure 14a indicate that soil moisture was not evenly distributed in the sandy slope model. Instead, the VWC values measured on profiles L and M indicate a general increase in soil moisture with increasing depth. On the other hand, a lower VWC value was measured for the soil in the near-surface zone. Achieving a homogeneous soil moisture profile in slope models made of clean sandy material proved difficult even for slope models with 30- and 40-degree inclinations. However, this was not the case for slopes made of SK10 and SK15 material (Figure 14b–d)), where a considerable amount of fine-grained clay fraction (kaolin) was added to the base soil, i.e., the uniformly graded sand. Figure 14b shows, for example, that the variations in the VWC values measured at the beginning of the test were much smaller in the SK15 soil than in the case of the clean sand, which indicates a more homogeneous distribution of soil moisture along all measurement profiles. The same applies to the SK10 material, i.e., Figure 14c,d). However, comparing Figure 14c,d, the homogeneity of the initial soil moisture distribution within the two slope models seemed to decrease with increasing soil water content and vice versa. The same was observed for all other soil types: even for a slope model built from clean sand, homogeneous conditions could be achieved if the soil moisture content was low enough.

All of the previously mentioned points seem to be closely related to the hydraulic properties of the soil, more precisely to the retention properties. Uniformly graded fine sand used as a base soil has a very steep SWRC with an AEV well below 1 kPa. With such a low AEV and a SWRC that covers a very narrow range between saturated and residual conditions (e.g., the soil approached residual conditions when matric suction values exceeded 4 to 5 kPa), the soil was not able to retain larger amounts of water within the soil structure, but the water tended to migrate to the lower part of the model (Figure 14a). Therefore, it was generally not possible to start the test on the slope model built in clean sand with a completely homogeneous distribution of soil moisture, as the redistribution of soil moisture took place almost immediately after the soil was placed in the flume and compacted. Only when the residual soil moisture was reached did the redistribution in the VWC of the soil cease and constant readings were observed over time on the TEROS 10 and 12 sensors.

This problem proved to be particularly relevant when a higher initial moisture content was required at the beginning of the test, as was the case with landslide models tested under seismic conditions or slope models built on a slope steeper than the base friction angle of the soil used as slope material. In the first case, it was found that slopes subjected to various seismic loads remained stable if the moisture content of the soil was not high enough. Due to the poor retention properties and relatively high permeability of the clean sand, the experiments could not be started with increased moisture content because in the period between the onset of rainfall and the start of the test (i.e., the application of the load by vibrating platforms), the redistribution of soil moisture would cause water to seep into the lower parts of the model, while the higher parts would remain at or near residual conditions. In the second case, i.e., when the stability of the downscaled slope model is ensured by the apparent cohesion that develops in partially saturated conditions (e.g., the case of the model built in clean sand with a 40-degree angle slope), maintaining the stability of the model implies keeping the VWC of the soil above residual conditions. Thus, gravity-induced redistribution of water within the slope model, as well as evaporation, requires additional wetting of the models during construction (compaction) and during the period between the completion of model build-up and the start of testing, i.e., the start of the rainfall simulation. Again, the occasional addition of water to the slope model and the redistribution of soil moisture in general led to an inhomogeneous distribution of water content in the slope models at the beginning of the tests.

**Figure 15:**
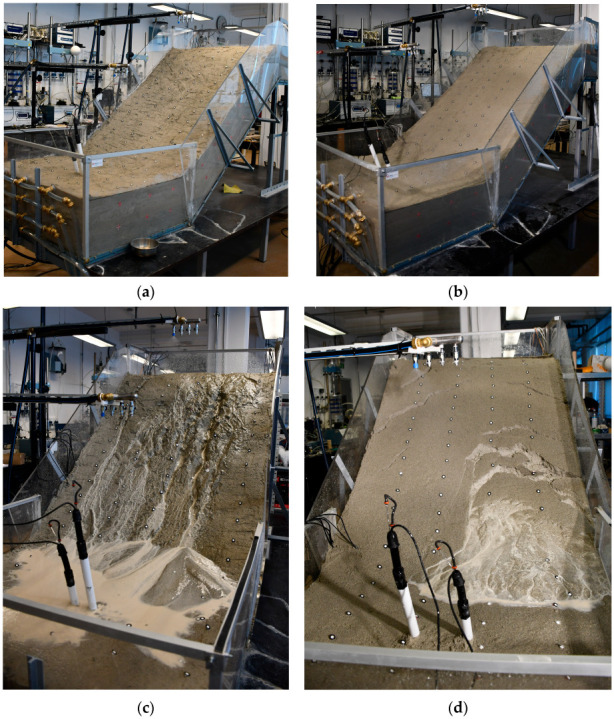
Two different types of instability depending on initial moisture in slope models: The slope model with higher initial moisture (**a**) leads to surface erosion (**c**), while the slope model with lower initial moisture (**b**) leads to the formation of cracks and landslides (d).

For sand-kaolin mixtures, the addition of fine-grained material resulted in lower hydraulic conductivity, but also increased the AEV of the soil and increased the area covered by SWRC between the saturated and residual states. The soil mixtures were thus able to retain a higher amount of water in the soil structure, while the redistribution process was significantly slowed down and mainly driven by the evaporation process rather than gravity, as was the case with the base soil material. Therefore, it was much easier to start the tests with homogeneous soil moisture distribution conditions in the slope models created with SK10 and SK15 materials. However, the problem with slope models made of sand-kaolin mixtures was excessive erosion, especially when the tests were carried out on slopes with a higher initial moisture. For models made of SK10 material with 35-degree angle slopes, one starting with a higher (Figure 14c) and one with a lower initial moisture content (Figure 14d), two different responses were observed in terms of the type of instability under the same rainfall conditions. As can be seen in Figure 15a, the surface of the model that started with an increase in soil moisture was eroded. In this case, however, no signs of cracking or the development of typical sliding surfaces were observed. On the contrary, when the slope model was built with a lower initial moisture content, typical cracks and the development of sliding surfaces were observed during the infiltration of rainfall.

Thus, if homogeneous distribution of soil moisture is a critical factor for the experiment to be conducted, the results of this study suggest that the targeted moisture conditions during model construction should be very low and close to residual conditions when slope models are constructed from relatively coarser and uniformly graded sandy soils with low retention and relatively high permeability. In contrast, the addition of a fine-grained fraction, i.e., the use of sand-kaolin mixtures, greatly facilitated the control of the initial soil moisture distribution in the slope models. In this case, however, the large overall volumetric changes that the soil may exhibit during rainfall infiltration, as well as excessive erosion, may pose serious limitations to the performance of small-scale physical landslide experiments on models built from such soil materials. To reduce these effects, it might be beneficial to subject downscaled slope models to several cycles of drying and wetting before starting the experiment and applying the desired rainfall conditions. In this way, the stiffness of the model due to over consolidation would be improved and the effects of the overall volume changes of the soil during rainfall simulation could be significantly reduced. On the other hand, it seems that using lower rainfall intensities and building the models with a lower initial moisture content would reduce surface erosion in the slope models.

## 5. Conclusions

This paper presented some of the results and experiences from the small-scale physical landslide model tests under 1 g conditions, obtained in the four-year research project “Physical modeling of landslide remediation constructions’ behavior under static and seismic actions”, which started in October 2018 at the University of Rijeka, Faculty of Civil Engineering, Croatia. The data were obtained from tests conducted on slope models created from different soil types, at different inclinations and initial moisture conditions, and for different simulated rainfall conditions. First, a brief description was provided of a newly developed platform that allows testing of downscaled slope models initiated by simulated rainfall, and its main components, including the rainfall simulator and the equipment used to monitor the hydraulic and mechanical response of the slope models during testing. The main considerations in the design of the flume and the selection of the monitoring equipment were described. Particular attention was given to the sensor network that allowed the monitoring of changes in soil moisture content and pwp conditions, both in terms of transient infiltration of rainfall through partially saturated soil and in terms of water table rise and pwp increase under fully saturated conditions. The main features of the sensors and measurement methods used to provide the measurement data were presented in more detail. Some advantages and disadvantages of the sensors used were described and some experiences about the suitability or possible limitations of using such devices for specific purposes and conditions envisaged in the project were discussed. Finally, three examples of the measurement results and interpretation of the data were given, which are among the many results obtained during the project activities. Data obtained with the hydrological monitoring equipment were discussed and analyzed in the context of rainfall-induced landslides, with the application to downscaled slope models subjected to controlled rainfall infiltration conditions.

The first example involved a model built in clean sand with a slope of 35 degrees and exposed to a constant rainfall intensity. Despite certain limitations, this example showed how monitoring changes in soil moisture can provide accurate (and apparently instantaneous) data on the increase in soil moisture content during different phases of transient rainfall infiltration, as well as on the subsequent GWL rise. The results presented were placed in the context of the initiation of a scaled sandy slope due to GWL rise and demonstrated how data on the observed hydraulic response and known initial and boundary conditions can be used as valuable tools for establishing relationships in a scaled slope model and investigating the conditions and mechanisms leading to slope failure.

The second example presented the hydraulic paths and stress states exhibited by the model built in SK15 material with a slope of 40 degrees during a simulated rainfall. Under certain working assumptions and using a simple theoretical approach, it was illustrated how principles of unsaturated soil mechanics can be implemented in the study of the response of small-scale physical landslide models and how the results obtained in such experiments can contribute to a better understanding of the driving mechanisms and to the quantification of the variables that govern the instability phenomena of rainfall-induced landslides. Data collected simultaneously at two measurement points during the experiment on changes in soil moisture and matric suction, plotted in the plane commonly used to represent soil water retention properties, showed that the two monitored points exhibited hydraulic hysteresis effects. Although this was not the aim of the experiment conducted, these results suggest another possible, very useful application of downscaled physical slope models—investigating the role of hydraulic hysteresis on HM response of unsaturated soil under conditions similar to those in a slope above the phreatic line, and its impact on slope stability under infiltrating rainfall conditions.

The third example summarized some observations related to possible difficulties in building the downscaled slope models under the targeted initial water content distribution conditions and discussed different types of instability observed in two tests as a function of initial soil moisture. The latter is often related to the antecedent rainfall conditions, which can make the difference between the occurrence or non-occurrence of landslides in a given rainfall event. The results presented suggest that in the case where a homogenous distribution of soil moisture is a condition for the test to be performed, the soil material should be installed with a relatively low target initial moisture content and closer to the residual moisture content. This is particularly true for highly permeable, coarse-grained soils, such as uniformly-graded sands, which typically have a low AEV and relatively steep SWRCs that cover a very narrow range of soil suction between the saturated and residual moisture contents. Although achieving the target initial moisture content was much easier for soil mixtures to which kaolin had been added, again the increased soil moisture conditions resulted in a less homogeneous distribution of moisture content along the measurement profiles. However, in contrast to the tests carried out on models made of clean sand, slopes made of sand-kaolin mixtures were affected by surface erosion and generally exhibited greater volumetric deformations when exposed to different rainfall conditions. The results suggest that the latter could be minimized by starting the tests with a lower soil moisture content, using lower rainfall intensities and/or increasing the preconsolidation pressure of the slope material by performing several drying and wetting cycles before starting the test and applying the desired rainfall characteristics.

The results presented in this paper are some of the preliminary findings of the project. Additional experiments specifically designed to investigate some of the issues raised and points discussed in this study need to be conducted to provide a more complete picture of how different variables affect the HM response of downscaled slope models and to advance the knowledge of the role they play in triggering rainfall-induced landslides in general. The results shown in this study suggest that the monitoring equipment and sensor network for monitoring the hydraulic response of small-scale physical landslide models adopted in the project are valuable and useful tools for such studies.

## Figures and Tables

**Table 1:** Basic soil properties of clean sand (S) and sand with 10% (SK10) and 15% (SK15) kaolin by mass at the target initial conditions.

Material	S	SK10	SK15
Specific gravity, *G_s_* (/)	2.70	2.69	2.67
Effective particle size			
*D*_10_ (mm)	0.190	0.038	0.056
*D*_60_ (mm)	0.370	0.310	0.207
Uniformity coefficient, *c_u_* (/)	1.95	8.16	54.11
Minimum void ratio, *e_mi_*_n_ (/)	0.64	0.65	0.54
Maximum void ratio, *e_max_* (/)	0.91	1.21	1.43
Hydraulic conductivity ^1^, *k_s_* (m/s)	1.0 × 10^−5^	6.8 × 10^−6^	3.5 × 10^−6^
Friction angle ^2^, *ϕ* (◦)	34.9	31.3	31.8
Cohesion ^2^, *c* (kPa)	0	3.9	4.4
Targeted initial porosity, *n_i_* (/)	0.44	0.47	0.43
Targeted initial relative density, *D_r_* (/)	0.5	0.5	0.75
Targeted initial water content, *w_i_* (%)	2	5	8.1

^1^ According to the preliminary results obtained with the constant and falling head test methods; ^2^ Based on the results of the direct shear tests.

**Table 2:** Technical characteristics of the full-cone nozzles used to simulate rainfall: spray angle and flow rates (v) for different working pressures (p) (lechler.com accessed on 12 February 2022).

Type	Spray Angle (°)		v (L/min)			
		p (bar)	1.0	2.0	3.0	5.0
490.404	60		0.76	1.00	1.18	1.44
490.444	60		0.95	1.25	1.47	1.80
490.484	60		1.21	1.60	1.88	2.31
490.524	60		1.52	2.00	2.35	2.89
460.524	90		0.30	0.40	0.47	0.58

## Data Availability

The data reported in the study can be obtained from the corresponding author on request.
